# Room temperature spin crossover properties in a series of mixed-anion Fe(NH_2_trz)_3_(BF_4_)_2−*x*_(SiF_6_)_*x*/2_ complexes†

**DOI:** 10.1039/d4dt00267a

**Published:** 2024-03-25

**Authors:** Xinyu Yang, Alejandro Enriquez-Cabrera, Kane Jacob, Yannick Coppel, Lionel Salmon, Azzedine Bousseksou

**Affiliations:** a LCC, CNRS & Université de Toulouse (UPS, INP) 31077 Toulouse France lionel.salmon@lcc-toulouse.fr azzedine.bousseksou@lcc-toulouse.fr

## Abstract

A series of mixed-anion Fe(NH_2_trz)_3_(BF_4_)_2−*x*_(SiF_6_)_*x*/2_ spin crossover complexes is obtained modifying the reaction time but also using an increase amount of tetraethyl orthosilicate as the source for the production and the incorporation of SiF_6_^2−^ competing with the BF_4_^2−^ anions present in the mother solution. The increase of the SiF_6_^2−^ anion inclusion to the detriment of the BF_4_^−^ counterpart induces a shift of the temperature transition toward high temperatures leading to interesting bistability properties around room temperature with *T*_1/2_ spanning from 300 K to 325 K. Moreover, the implementation of a solid–liquid post synthetic modification approach from the Fe(NH_2_trz)_3_(BF_4_)_2_ parent complex with identical TEOS proportions and under certain experimental conditions lead systematically to the same Fe(NH_2_trz)_3_(BF_4_)_1.2_(SiF_6_)_0.4_ composition. This compound presents an abrupt spin crossover behaviour with a narrow hysteresis loop just above room temperature (320 K), which is stable under thermal cycling and along time with no specific storage conditions. Such crystalline powder sample incorporates homogeneous rod-shaped particles whose formation and physical properties can be followed simultaneously using infra-red spectroscopy, dynamic light scattering (DLS), transmission electronic microscopy (TEM) and optical reflectance. The observation of a stabilized single *ca.* 800 nm population of mixed-anion particles starting from insoluble various sizes (from nano- to microscale) Fe(NH_2_trz)_3_(BF_4_)_2_ particles supports the key role of the solvent (water molecules) on the separation, the reactivity and the reorganization of the 1D iron-triazole chains forming the packing of the structure.

## Abstract 2

A series of mixed-anion Fe(NH_2_trz)_3_(BF_4_)_2−*x*_(SiF_6_)_*x*/2_ complexes presenting abrupt spin crossover behaviour with a narrow hysteresis loop just above room temperature is obtained by coordination reaction or solid–liquid post synthetic modification.

## Graphical abstract

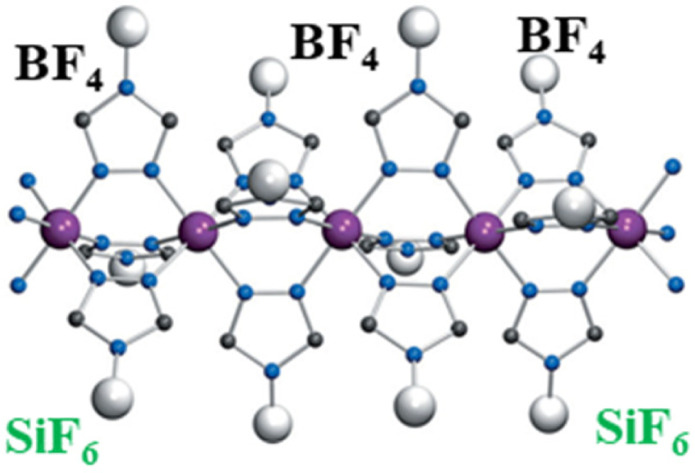

## 1. Introduction

Molecular spin crossover (SCO) complexes are phase change materials that exhibit variable magnetic and optical properties associated with a significant volume change upon a stimulus like temperature variation and have been proposed as active elements for photonic, electronic and mechanical devices.^1–3^ Although numerous examples of SCO behavior spanning from 80 to 500 K have been reported in the literature for different 3d metals,^4–11^ there is still an expectation to obtain stable and integrable compounds with transition temperatures just above room temperature, with or without hysteresis loop to confer or not a memory effect in order to target functional applications. It is generally accepted that controlling the spin state in metal complexes is still challenging because of the difficulty to foresee the structural properties of the secondary coordination sphere and the concomitant structural/SCO properties relationships. One of the most studied class of iron(ii) compounds belongs to the Fe-triazole complex family with iron ions developing along 1D chains including three neutral triazole bridging ligands; where the positive charge of the metal centers is counterbalanced by the presence of counter anions interspersed between the chains.^12,13^ The hysteretic spin crossover behavior generally observed for such complexes is explain by the presence of a strong intrachain cooperativity, coupled with interchain supramolecular interactions.^12^ One of the strategies developed to modify the spin transition temperature in this family of compounds consisted of partially substituting either the ligand or the metal within coordination complexes.^14–17^ In particular, the dilution of iron with SCO inactive metal (zinc) complex led to the decrease of the temperature transition as well as the reduction of the cooperativity between iron centers affording reduced-width hysteresis and/or conversion with more gradual characters. This ‘molecular alloying’ approach has been explored both with bulk and nanosized SCO materials.^18–21^ In general, the approach to substitute the selected element (metal center or ligand) consisted simply to mix the corresponding precursors, with a specific quantity ratio to modulate the final chemical composition of the product. In fact, no clear result was reported concerning the synthesis of a corresponding pure mixed counter-anion derivative. Recently, Vinogradova and collaborators reported the synthesis of the Fe(NH_2_Trz)_3_(NO_3_)_2(1−*x*)_(SO_4_)_*x*_ derivatives but the SCO behavior in two steps with transition temperature as those of the two parents compounds seems to suggest that it is a physical mixture and not a mixed counter-anion complex.^22^ In the same way, a powder mixture of Fe(NH_2_trz)_3_(BF_4_)(SiF_6_)_0.5_ and Fe(NH_2_trz)_3_(BF_4_)_2_ complexes was reported showing the occurrence of a two-steps spin transition corresponding to the two independent properties for each complex.^23^ In this case, the hardly controllable anion substitution can be explained by the ability of the BF_4_^−^ anion to dissociate into hydrofluoric acid, the latter reacting with the glass tube (SiO_2_) to form the SiF_6_^2−^ anions, which are then in competition with the BF_4_^−^ anions in the solution during the formation of the Fe(NH_2_trz)_3_(BF_4_)_2_ complex. Moreover, X-ray diffraction study revealed the crystal structure of Fe(NH_2_trz)_3_(BF_4_)(SiF_6_)_0.5_.^23^ Our group and others have used tetraethyl orthosilicate (TEOS) as a SiO_2_ precursor in reverse micelle syntheses to elaborate functionalized core@shell [Fe(Htrz)_2_(trz)](BF_4_)@SiO_2_ nanoparticles.^19,24–28^ In the latter cases, the exchange of the BF_4_^−^ by the SiF_6_^2−^ was not observed revealing certainly the key role play by the NH_2_trz ligand in the substitution mechanism. Shepherd and collaborators recently used a solid–solid post synthetic modification (PSM) grinding method to fully substitute halogen counter-anions in similar iron(ii) 1,2,4-triazole based spin crossover materials.^29^ On the other hand, solid–liquid^30^ and solid–vapor^31–34^ post synthetic modifications have been also reported for this family of complexes. In particular, we have demonstrated that playing with the experimental conditions, both partial but more importantly complete solid–liquid PSM can be obtained from the [Fe(NH_2_trz)_3_](NO_3_)_2_ derivative by reaction on the ligand with different substrates.^35^ It was shown that some iron(ii) complexes not achievable by the classic synthesis method can be obtained by the PSM approach. In a recent paper, we have also reported that both conventional and PSM method can be efficient to obtain a pure mixed-anion [Fe(NH_2_trz)_3_](BF_4_)_1,2_(SiF_6_)_0.4_ complex when TEOS is used as SiO_2_ precursor.^36^ In fact, there is good reasons to think that the SCO properties of a complex with similar composition were also reported unknowingly by Díaz-Ortega and collaborators.^37^ Indeed, it would appear that they are mistakenly attributing the change in transition temperature to a matrix effect in {[Fe(NH_2_trz)_3_](BF_4_)_2_}@SiO_2_, when the origin of this variation is certainly the composition of the complex. Moreover, the same authors did not report such matrix effect in a former paper for similar complexes.^19^ The aim of the present report is to complete our study and probe if the composition of the mixed anion Fe(NH_2_trz)_3_(BF_4_)_2−*x*_(SiF_6_)_*x*/2_ complexes can be varied while modifying the quantity of TEOS reagent, the reaction time and the reaction temperature in order to try to better control the transition temperatures of such complexes around room temperature for targeted applications.

## 2. Experimental section

### 2.1. Sample characterization

NMR experiments were recorded on a Bruker Avance 400 III HD spectrometer operating at magnetic fields of 9.4 T. Samples were packed into 3.2 mm or 4 mm rotor and spun at different spinning rate (typically between 9 to 18 kHz) at low temperature (external temperature of 225 K or 231 K). ^19^F magical angle spinning (MAS) solid state nuclear magnetic resonance (ssNMR) were measured with Hahn-echo scheme synchronized with the spinning rate and relaxation delay of 10 s. ^29^Si CP (CP = cross-polarization) MAS spectra were recorded with a recycle delay of 1.5 s and a contact time of 3 ms. ^29^Si MAS were acquired with single pulse experiments with recycle delays of 3 s. Chemical shifts were externally referenced to CCl_3_F and tetramethylsilane for ^19^F and ^29^Si, respectively.

The size and morphology of the SCO particles were determined by transmission electron microscopy (TEM) using a JEOL JEM-1011. TEM samples were prepared by placing a drop of the particles (suspended in ethanol) on a carbon-coated copper grid. DLS measurements were performed using the Malvern Zetasizer Nano ZS. Variable-temperature optical reflectance data were acquired with a MOTIC SMZ-168 stereomicroscope equipped with MOTICAM 1000 color CMOS camera. A 2 K min^−1^ rate was used for both cooling and heating. Powder X-ray diffraction patterns were recorded using a PANalytical X'Pert equipped with a Cu X-ray tube, a Ge(111) incident beam monochromator (*λ* = 1.5406 Å) and an X'Celerator detector. FTIR spectra were recorded at room temperature with a PerkinElmer Spectrum 100 spectrometer in ATR mode (resolution *ca.* 1 cm^−1^) between 650 cm^−1^ and 3500 cm^−1^.

### 2.2. Synthesis of the spin crossover complex

All reagents were purchased from Sigma Aldrich and used without further purification. Molar equivalent of TEOS is given *versus* the iron salt. Sample 1a was synthesized by dissolving 0.2 g of Fe(BF_4_)_2_·6H_2_O in 2 mL of water in a plastic flask and adding successively 0.0155 g (0.125 eq.) of TEOS and 0.149 g (3 eq.) of 4-NH_2_-1,2,4-triazole. The suspension was stirred overnight (15 hours) and the product purified by three successive ethanol washing/centrifugation cycles resulting in 0.120*g* of a pink solid (41% yield). Sample 1b–1d were obtained in similar experimental conditions using 0.25 eq. (0.031 g), 0.5 eq. (0.062 g) and 1 eq. (0.123 g) of TEOS, resulting in 0.180 mg (62% yield), 0.205 mg (72% yield) and 0.245 mg (76% yield) of complex, respectively. Samples 1e–1g were synthesized with 0.3 eq. (0.036 g) of TEOS with increasing reaction time of 2 hours, 15 hours and 3 days, resulting in 0.200 mg (69% yield), 0.190 mg (65% yield) and 0.220 mg (76% yield) of complex, respectively. [Fe(NH_2_trz)_3_](BF_4_)_2_ (2) was characterized in ref. 36. Concerning the PSM reaction, 0.133 g of 2 was suspended in 1 mL of water at room temperature (298 K) and 0.017 g (0.3 eq.) of TEOS was added to synthesize sample 3a. The white suspension was stirred at room temperature for 4 days, showing a progressive color change from white to pink. Sample 3b and 3c were synthesized as 3a with increasing quantity of TEOS: 0.034 g (0.6 eq.) and 0.068 g (1.2 eq.), respectively while sample 3d was obtained after 7 days of reaction and sample 3e and 3f increasing the temperature of reaction at 323 K and 343 K, respectively. Sample 3d and 3e are pure mixed anion samples leading after three successive ethanol washing/centrifugation cycles to 0.093*g* (72% yield) and 0.097*g* (75% yield), respectively.

## 3. Results and discussion

**Fig. 1:**
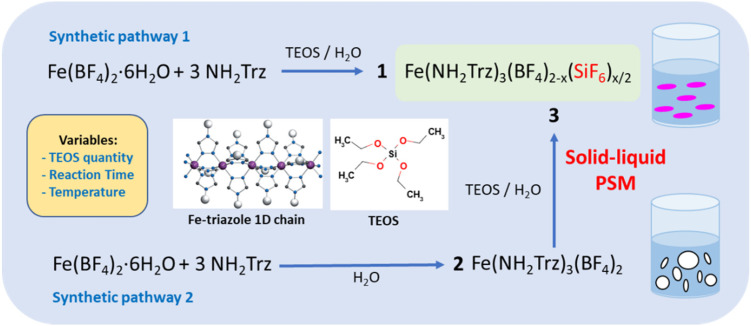
Reaction scheme and representation of the 1D “Fe-triazole” chains.

As shown in Fig. 1 for both “classic” coordination reaction and solid–liquid post synthetic modification method and in order to obtain pure powder sample of the mixed-anions Fe(NH_2_trz)_3_(BF_4_)_2−*x*_(SiF_6_)_*x*/2_ complex, a controlled quantity of TEOS was used as SiF_6_^2−^ precursor and the reactions were performed in a plastic container to avoid competition with the SiO_2_ ripped from the wall of the glass container.

Concerning the first synthetic pathway, a series of complexes 1a–1g was obtained modifying both the concentration of TEOS from 0.25 to 1 eq. *vs*. iron and the reaction time from 2 hours to 3 days according to Table 1. The synthesis of the preliminary complex [Fe(NH_2_trz)_3_](BF_4_)_2_ (2) was already reported and the white color of the obtained solid agrees with an HS state for the iron(ii) center.^38^ Based on the synthetic pathway 2, a series of complexes 3a–3f was obtained following a post synthetic modification approach by reaction of the solid complex 2 in an aqueous solution of TEOS. In this case, the quantity of TEOS from 0.3 to 1.2 eq. *vs*. iron but also the reaction time up to 7 days and the reaction temperature from 298 to 343 K were varied.

**Table 1:** Experimental conditions, formulae extracted from ^19^F and ^27^Si NMR and transition temperatures taken from optical reflectance measurements for all synthesized samples

Sample	*T* (K)	TEOS	Time	Formulae from ^19^F and ^29^Si NMR	*T* ^↓^_1/2_	*T* ^↑^_1/2_
1a	298 K	0.125 eq.	15H	[Fe(NH_2_trz)_3_](BF_4_)_1.46_(SiF_6_)_0.27_	300	309
1b	298 K	0.25 eq.	15H	[Fe(NH_2_trz)_3_](BF_4_)_1.31_(SiF_6_)_0.34_	307	313
1c	298 K	0.5 eq.	15H	[Fe(NH_2_trz)_3_](BF_4_)_1.20_(SiF_6_)_0.40_·0.04SiO_2_	313	316
1d	298 K	1 eq.	15H	[Fe(NH_2_trz)_3_](BF_4_)_1.27_(SiF_6_)_0.36_·1.01SiO_2_	315	317
1e	298 K	0.3 eq.	2H	[Fe(NH_2_trz)_3_](BF_4_)_1.45_(SiF_6_)_0.27_	298	307
1f	298 K	0.3 eq.	15H	[Fe(NH_2_trz)_3_](BF_4_)_1.25_(SiF_6_)_0.38_	319	326
1g	298 K	0.3 eq.	3D	[Fe(NH_2_trz)_3_](BF_4_)_1.26_(SiF_6_)_0.37_·0.07SiO_2_	316	322
2	—	—	—	[Fe(NH_2_trz)_3_](BF_4_)_2_	230	240
3a	298 K	0.3 eq.	4D	Mixture of complex	318^a^	326^a^
3b	298 K	0.6 eq.	4D	Mixture of complex	318^a^	325^a^
3c	298 K	1.2 eq.	4D	Mixture of complex	316^a^	322^a^
3d	298 K	0.3 eq.	7D	[Fe(NH_2_trz)_3_](BF_4_)_1.21_(SiF_6_)_0.40_	316	326
3e	323 K	0.3 eq.	4D	[Fe(NH_2_trz)_3_](BF_4_)_1.17_(SiF_6_)_.0.42_	317	328
3f	343 K	0.3 eq.	4D	b	248	255

a Transition temperatures for the mixed complex.

b Not determined as the complex is paramagnetic for our experimental boundary conditions.

The composition of all complexes was determined by solid state nuclear magnetic resonance (ssNMR) with the magical angle spinning (MAS) technique.^39,40^ However, MAS spinning results to a temperature increase of the sample because of the friction between the air and the rotating rotor which implies that measurements at low temperature have to be performed in our case to keep the iron complex diamagnetic. According to this approach, we have previously demonstrated that such technique can give a good enough composition as precise as that obtained by elemental analyses.^36^

**Fig. 2:**
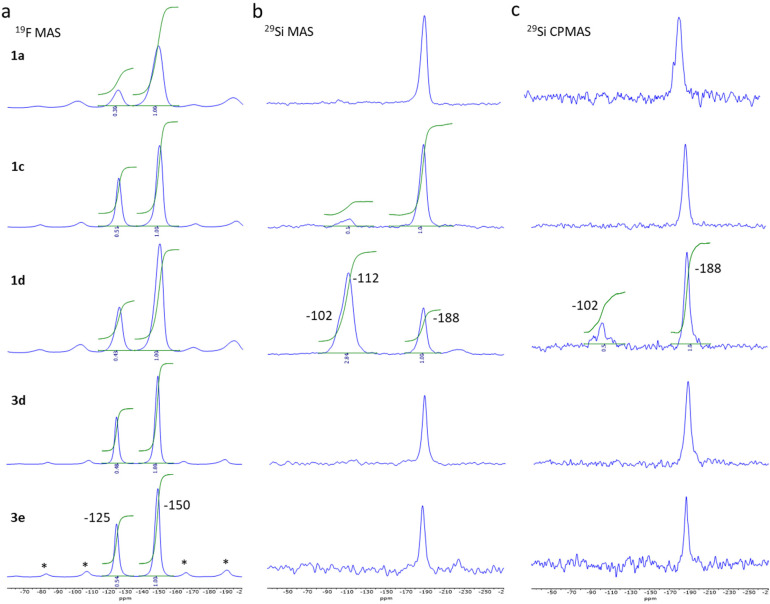
^19^F MAS ssNMR spectra obtained at 228 K and *ν*_r_ of 18 kHz, *: spinning sidebands (a), ^29^Si MAS (b) and CPMAS (c) spectra obtained at 231 K and *ν*_r_ of 9 kHz for sample 1a, 1c, 1d, 3d and 3e.

Fig. 2 reports ^19^F Hahn-echo and ^29^Si MAS SSNMR spectra for spinning frequencies (*ν*_r_) of 18 and 9 kHz and at low temperature, 225 and 231 K, respectively for selected samples from the two series of syntheses (see also Fig. S1–S4† for complementary data). Such experimental conditions are good enough to clearly separate the isotropic signals of the two signals from their sidebands associated to chemical shift anisotropy while removing the magnetic interactions between nuclear and electron spins in the case of paramagnetic species.

The ^19^F MAS Hahn-echo spectra exhibit clear isotropic resonance corresponding to the BF_4_^−^ and SiF_6_^2−^ anion located at −150 ppm and −125 ppm (Fig. 2a), respectively, in agreement with earlier studies.^41,42^ Separation of resonance allowed us to obtain good integrations and the corresponding SiF_6_^2−^/BF_4_^−^ intensity ratio, which varies, from 0.30 ± 0.02 to 0.54 ± 0.02 considering the two series of experiments.

For sample 1a and 1b (0.125 and 0.25 eq. TEOS, respectively), both ^29^Si MAS (Fig. 2b) and ^29^Si CPMAS (Fig. 2c) spectrum exhibit only one peak at −188 ppm, which can be attributed to the SiF_6_^2−^ anion. The observation of the peak for the ^29^Si CPMAS is associated with ^1^H → ^19^Si dipolar transfer between the hydrogens of the triazole ligands and the silicon of the SiF_6_^2−^ anions.^43,44^ For sample 1c (0.5 eq. TEOS), a second peak is also observable at −112 ppm but only for the MAS spectrum. This resonance attributed to Q^4^ siloxane bridge (SiO)_4_Si is compatible with the assumption of a core–shell structure with the SCO complex as the core and the SiO_2_ as a thin shell. On the contrary, for sample 1d (1 eq. TEOS), an additional resonance at −102 ppm appears both in the ^29^Si MAS and ^29^Si CPMAS spectrum and corresponds to Q^3^ single silanol (SiO)_3_SiOH. The presence of Q^4^ and Q^3^ resonances agrees with the formation of additional SiO_2_ nanoparticles including a shell of silanol, as confirmed by the transmission electronic microscopy images (see Fig. S11†). Same observations were made for the sample 1g synthesized in three days with 0.3 equivalent of TEOS. Thus, an increase of the TEOS quantity or the reaction time lead at some level to the formation of undesirable SiO_2_ nanoparticles. For pure samples of the series 3, similar peak can be observed at −188 ppm (^19^F) for the SiF_6_^2−^ anion but only traces (unquantified) of SiOH and SiO_2_ are possibly detected for sample 3d (see infrared spectroscopy study hereafter).

It can also be noted that the ^19^F and ^29^Si signals of compounds from series 1 are broader than those of series 3 due to structural/chemical heterogeneities. This can be related to a less homogeneous composition and morphology and possibly the presence of a small amorphous phase explaining the disordered spin crossover behaviour (*vide infra*).

All the NMR data were combined to propose a chemical composition for all synthesized compounds, which is given in Table 1. For the bulk synthesis series and up to a certain limit both the quantity of TEOS and the reaction time allow to increase the SiF_6_^2−^ anion inclusion amount. Nevertheless, and in agreement with the comparison of the spin crossover properties for the different samples, shown hereafter, the increase of the quantity of TEOS beyond 0.5 eq. and the reaction time beyond 15 hours do not increase further the insertion of SiF_6_^2−^ anions. For the PSM series (samples 3a–3h), 4 days of reaction was not sufficient to transform all the starting complex 2 into a mixed anion complex regardless of the amount of TEOS used. For 0.3 eq. of TEOS (3d), the total reaction of complex 2 was reach after 7 days leading to a final composition similar to sample 1f. It is interesting to note that the increase of the quantity of TEOS for a 7 days reaction did not increase the SiF_6_^2−^ insertion. The same reaction as 3a carried out at 323 K (sample 1g) was completed in only 4 days while a further increase of temperature to 343 K afforded a non-expected composition at a first glance. In fact, at such high temperature, the complex is slightly soluble and the mechanism involved in the formation of the mixed complex is different (*vs*. PSM) leading to a reduced insertion of the SiF_6_^2−^ anions.

**Fig. 3:**
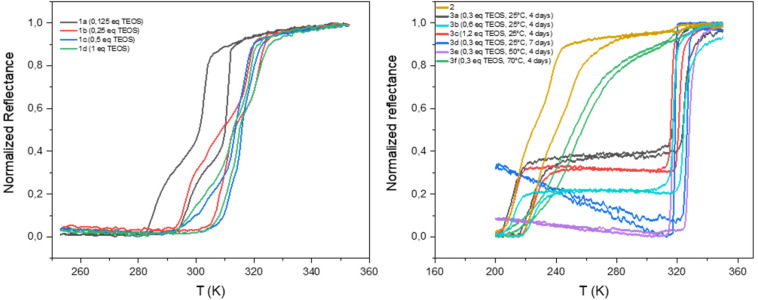
Thermal variation of the optical reflectance (desolvated sample) in the heating and cooling mode for samples 1a–1d, 2 and 3a–3f.

The homogeneity and the spin crossover properties of the sample of both series were probed by optical reflectance (see Fig. 3). Transition temperatures for all samples corresponding to the second thermal cycle are gathered in Table 1 with the chemical composition. For the first series of complexes (conventional synthesis in presence of TEOS), the profile of the thermal variation of the optical reflectance is gradual, asymmetric and stepped. This behavior can be related to distinct crystalline phases and/or the presence of defects in the structure in line with the rather fast precipitation of the compound. The increase of the TEOS equivalent from 0.125 to 0.5 eq. (*versus* iron) tends to increase the transition temperatures while a further increase to 1 eq. does not appear to have any significant consequence. The relationship in between composition and transition temperatures indicates that the increase of the transition temperatures is in line with the mixed anion composition and the increase of the SiF_6_^2−^ insertion.

As shown in Fig. S5,† similar tendencies can be obtained while playing with the reaction time. It seems that the transitions temperature can be controlled by modifying the reaction time in the 2–12 hours’ time slot whereas longer times seem to have no effect. Thus, the transition temperature can vary from 300 to 325 K. The controllability of these syntheses was proven by the repetition of the same synthesis affording similar composition and spin crossover behavior. It is interesting to note that no experimental condition used permitted to remove the non-monotonous behavior. Fig. S6† shows also that the spin crossover properties is stable upon consecutive thermal cycling and that the [Fe(NH_2_trz)_3_](BF_4_)_2_ complex (2) is not formed whatever the experimental conditions used.

As already reported, the starting compound 2 implemented for the post synthetic modification approach exhibits a multistep spin transition centered at *ca.* 235 K with a 10 K hysteresis loop.^39^ This behavior was associated with the presence of distinct crystalline phases. Thermal variation of the optical reflectance for the second series of experiments (post synthetic modification) is also reported for all samples in Fig. 3. In the first tries, experiments were conducted at room temperature and the quantity of TEOS was increased from 0.3 to 1.2 eq. for a reaction time of 4 days. Although, quantitative comparison of the relative proportion of the starting and post synthesized complexes is not accurate (using the optical reflectance of different samples), we can estimate that the increase of the quantity of TEOS doesn't seem to be a primary factor for the control of the SiF_6_^2−^ anion inclusion and the associated increase of the transition temperatures. In contrast to the first series of experiments, the PSM approach does not permit to control the transition temperatures of the complex; only one composition/spin crossover properties is obtained mixed or not with the starting material. In fact, 7 days of reaction was necessary to complete the modification of sample 2 into a mixed anion complex. Such result can also be obtained in 4 days when the synthesis is carried out at 323 K while further increase of temperature to 343 K led to reduce insertion of the SiF_6_^2−^ anion certainly in an inhomogeneous manner leading to a gradual behavior at lower temperature compared to other mixed anion complexes of the series.

Although samples of series 1 and 3 present similar temperature transition, in line with the close chemical composition, the behavior is drastically diverse. Indeed, the profile of the thermal variation of the optical reflectance for series 3 shows, for the desolvated form, a first order abrupt transition with a narrow hysteresis loop with *T*^↑^_1/2_ of 316 K and *T*^↓^_1/2_ of 325 K.

It appears interesting to compare the temperature transitions of such mixed-anion complexes with the corresponding non-mixed parent ones (Fe(NH_2_Trz)_3_(SiF_6_) and Fe(NH_2_Trz)_3_(BF_4_)_2_). Surprisingly, the reported SCO behavior of the Fe(NH_2_Trz)_3_(SiF_6_) complex with transition temperature around 250 K (ref. 45) is not in line with the general tendency observed for the mixed-ligand and mixed-metal counterparts for which increasing ligand and metal exchange led to a concomitant and progressive shift of the temperature transitions in between those of the two parent complexes.^14–17^

**Fig. 4:**
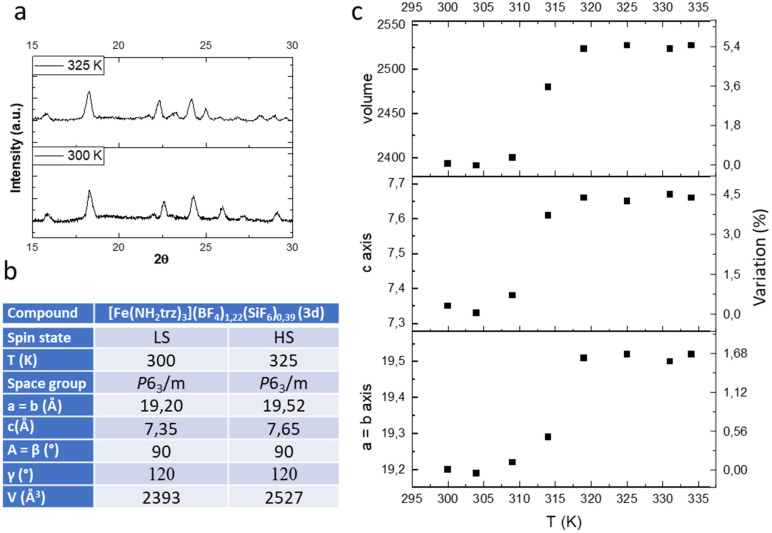
Representative powder X-ray diffraction pattern in the LS and HS states (a), unit-cell parameters (b) and thermal variation of the unit cell parameters (c) of complex 3d.

In the previous study we have shown by powder X-ray diffraction (PXRD) that both samples obtained by conventional or PSM methods are isostructural but the homogeneity and the crystallinity is higher for samples obtained by PSM explaining the non-monotonous character of the spin transition for sample of series 1 in agreement with the composition and the presence of defects in the structure and/or distinct crystalline phases. In the present study, we have also performed a series of variable-temperature PXRD experiments on polycrystalline sample 3d (a pure sample obtained by PSM). Selected part of representative diffractograms in the two spin states are shown in Fig. 4a, and the main structural parameters are summarized in the inserted Table (see also Fig. S7† for a compilation of the ensemble of the experimental data).

As expected, the transition from the LS to the HS form is accompanied by a significant expansion of the unit cell volume (Fig. 4c). Compound 3d expands its volume by nearly 6% while the ordinary thermal dilation seems to be rather weak. Importantly for mechanical investigation and application as actuators, highly anisotropic transformation strains have been found in the compound. The largest elongation of *ca.* 5.5% occurs in the direction of the chains along the parameter *c* while less than 2% elongation is obtained for the two other directions *a* and *b*.

**Fig. 5:**
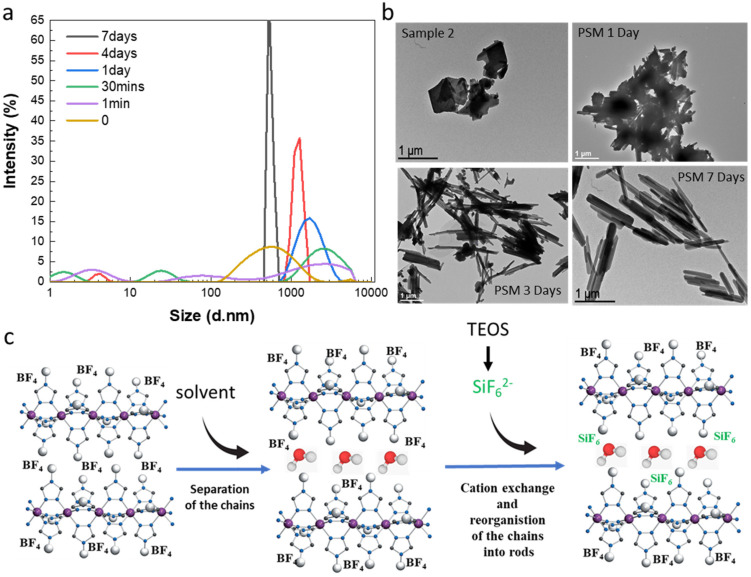
Dynamic light scattering measurements (a) and selected transmission electronic microscopy images (b) over time for PSM syntheses and proposed mechanism for the formation of the mixed anions complex rods (c).

By observing images obtained from transmission electronic microscopy (TEM), we reported that as expected for the synthesis of bulk samples, rather inhomogeneous microparticles with no particular shape are obtained for samples 1f and 2 (Fig. 5b). Similar results are obtained for all samples belonging to synthesis series 1 (see Fig. S11†). Surprisingly starting from the micrometric platelet objects in 2, the anion exchange PSM reaction afforded homogeneous rod like particles with less than 1 μm length (Fig. 5b and ref. 36).

In order to complete the study and to understand the mechanism involved in the formation of the mixed anion complex rods, simultaneous dynamic light scattering (DLS), infrared spectroscopy, NMR, TEM and optical reflectance measurements were performed over the time.

Experimental conditions for the synthesis of this new series of samples are similar to those used for the synthesis of sample 3a and 3d (0.3 eq. TEOS, room temperature). DLS measurements as a function of time do show the presence of inhomogeneous micrometric objects in fresh suspension of 2 (*t* = 0) and their evolution into more regular *ca.* 700 nm micro-structures after 7 days in agreement with the observation of the homogeneous rods by TEM (Fig. 5a and b). For intermediate times, we observe in a first instance the appearance of various size populations with an increasing size for the largest population and then a general decrease of the size and the concomitant homogenization of the size of the particles. These observations agree with the evolution of the TEM images upon time showed in Fig. 5b (see also all data given in Fig. S12†). In particular, the TEM image taken after 1 day of reaction reveals the presence of agglomerated particles including rod-shaped ones while after 3 days of reaction and although smaller size particles can be observed, we can clearly see in majority the rod particles.

Complementary infrared spectroscopy and optical reflectance measurements performed in parallel and shown in Fig. S8† confirm the increase of the substitution of the BF_4_^−^ anions by the SiF_6_^2−^ anions over time leading to a pure mixed anion complex following 7 days of reaction. Indeed, infrared spectroscopy shows the characteristic vibration modes of the different counter anions. For sample 2, an intense band attributed to the BF_4_^−^ anions is observed at 1020 and 1095 cm^−1^. The intensity of these vibration modes decreases with the associated appearance of the *ν*3 stretching vibration band at 730 cm^−1^ and the *ν*4 bending vibration band at 470 cm^−1^ characteristics of the SiF_6_^2−^ ion.^46^ Moreover, as suggested by the ^29^Si NMR spectrum of sample 3d, the signature of the presence of SiO_2_ is observable, in particular for the final product, with bands located at 1080 cm^−1^ and 780 cm^−1^ corresponding to the Si–O–Si and Si–O stretching vibrations, respectively.

As shown also in Fig. S8,† the evolution of the spin crossover properties extracted from the thermal variation of the optical reflectance for that new series of samples compares well with that obtained for the previous 3a and 3e samples, the transition temperatures being slightly shifted towards higher temperatures in the latter cases maybe due to residual water molecules in the complexes. Fig. S9 and S10† show also the stability of the complex obtained by PSM upon thermal cycle and over time, respectively.

All these data have come together to propose a mechanism to explain the completeness of the solid–liquid PSM reaction for an apparently non-porous material and the formation of the rod for the resulting mixed anion complexes (Fig. 5c). Such surprising morphological reorganization was already observed for similar solid–liquid PSM reaction in this family of compounds.^35^ Present results agree also with the assumption, which implies that water as polar solvent, thanks to specific interactions with the BF_4_^2−^ counter-anions localized in between the Fe-triazole chains, can initially favor the separation of the latter decreasing the size of the crystallites/particles and promoting the anion exchange. This hypothesis is also supported by the quite important distance between neighboring chains of 11.34 Å reported for the parent complex 2.^23^ Thus in a second step, the reduce size objects can be the site of a solid–solid reorganization/restructuration leading to more stable rod-shaped particles.

## 4. Conclusion

Using TEOS precursor as source of SiF_6_^2−^ counter-anions together with the tetrafluoroborate iron salt, a series of pure mixed anion Fe(NH_2_trz)_3_(BF_4_)_2−*x*_(SiF_6_)_*x*/2_ complexes were synthesized according to a classical coordination chemistry reaction. Although their spin crossover properties exhibit non-monotonous spin transition certainly due to the presence of defects or crystalline domains related to a fast precipitation, they present also stable transition temperature just above room temperature (300–325 K), which remains quite rare in the field. In contrast, solid–liquid post-synthetic modification (PSM) approach from the solid Fe(NH_2_trz)_3_(BF_4_)_2_ complex realized in a solution of TEOS permitted to obtain only one pure composition Fe(NH_2_trz)_3_(BF_4_)_1.2_(SiF_6_)_0.4_. This compound on the other hand presents a singular abrupt spin crossover with a narrow (less than 10 K) hysteresis loop centred just above room temperature (320 K), which makes this compound rather unusual and adequate for its integration into devices for future various applications. The complete transformation of the parent complex is obtained in 4–7 days depending on the reaction temperature and lead to a more homogeneous and crystalline sample (*versus* classical syntheses) containing regular rod-shaped particles. Powder X-ray diffraction measured over temperature revealed the significant volume change of the unit cell (*ca.* 6%) upon spin crossover and the anisotropy of the variation meanly along the direction of the chains. The monitoring of the PSM reaction upon time by the combination of dynamic light scattering, infrared spectroscopy, transmission electronic microscopy and optical reflectance confirms the proposed mechanism, which can explain the transformation in solid state from the micrometric unshaped particles of the parent compound to the regular anisometric particles for the final mixed anion complex. It is suggested that (1) the solvent induces the partial separation of the 1D chains of the complex favorizing the anion exchange and the formation of the mixed anion complex and (2) the resulting reduced size of matter can reorganized itself leading to structurally more stable rod-shaped particles. Eventually, the convenient transition temperature of this SCO complex, associated with the anisometric morphology of the particles that it forms, suitable for their organisation/alignment into films and showing a relevant volume change with an anisotropic character makes this scarce, quasi-ideal and stable material promising for real application for instance as active element in molecular actuators.^47^

## Author contributions

Samples fabrication and characterization (X. Y., A. E. C. and K. J.), NMR study (Y. C.), physical property measurements (L. S., A. B.). Paper writing (L. S.). All authors contributed to the data analysis

## Conflicts of interest

There are no conflicts to declare.

## Supplementary Material

DT-053-D4DT00267A-s001
