# Supplementary material for: Room temperature spin crossover properties in a series of mixed-anion Fe(NH2trz)3(BF4)2−x(SiF6)x/2 complexes
Source: Dalton Trans. 2024 Mar 25;53(15):6830–8. doi: 10.1039/d4dt00267a (PMC11019404; doi:10.1039/d4dt00267a)

This journal is © The Royal Society of Chemistry 2024

Room temperature spin crossover properties in a series of mixed-anion $Fe(NH_2trz)_3(BF_4)_{2-x}(SiF_6)_{x/2}$ complexes

Xinyu Yang, Alejandro Enriquez Cabrera, Kane Jacob, Yannick Coppel, Lionel Salmon, Azzedine Bousseksou**

LCC, CNRS & Université de Toulouse (UPS, INP), 31077 Toulouse, France

* lionel.salmon@lcc-toulouse.fr, azzedine.bousseksou@lcc-toulouse.fr

Figure S1: ^{19}F MAS ssNMR spectrum for sample **1a-1g** (*: spinning sidebands)

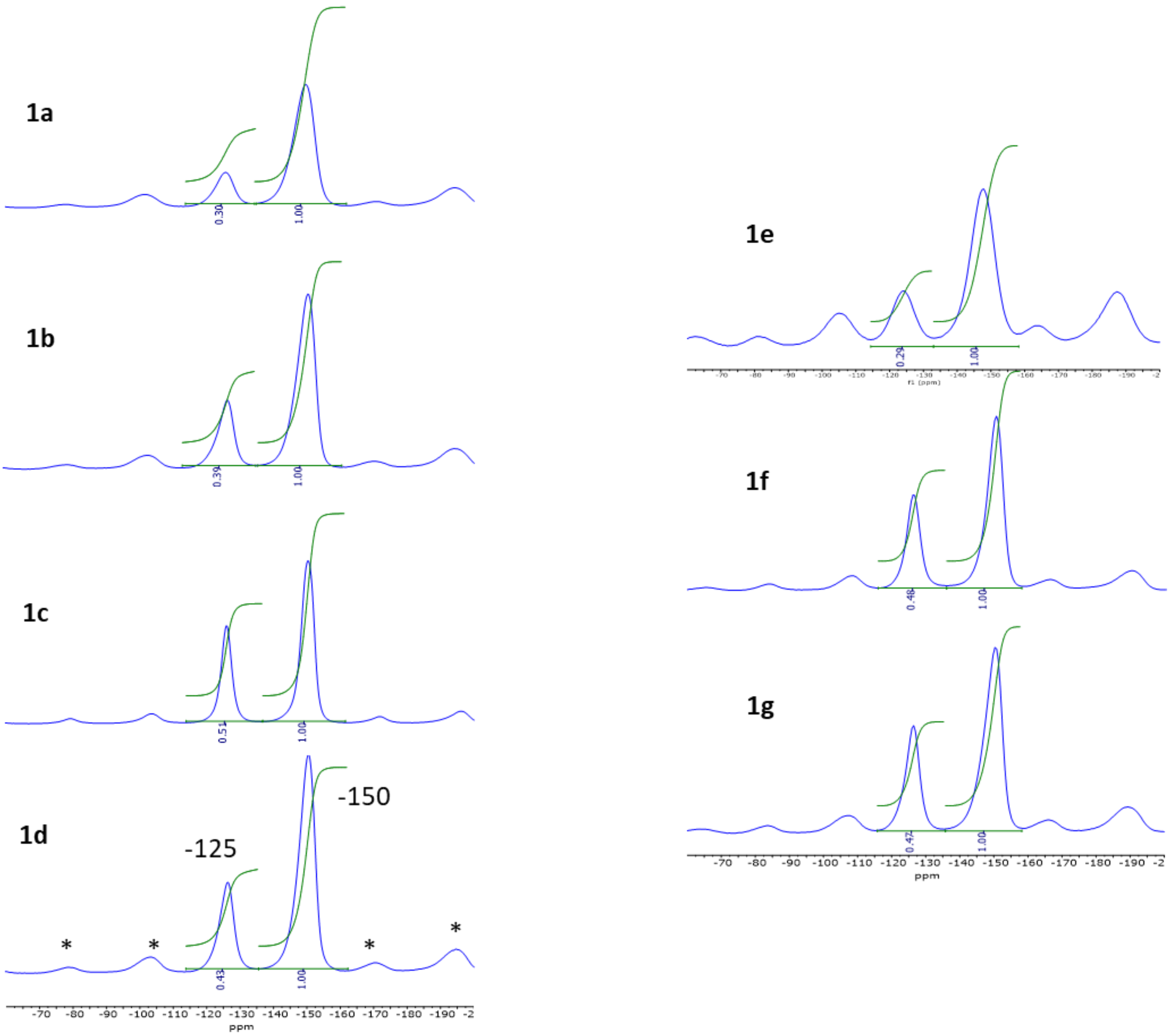

Figure S2: ^{19}F MAS ssNMR spectrum for sample **3d-3f** (*: spinning sidebands)

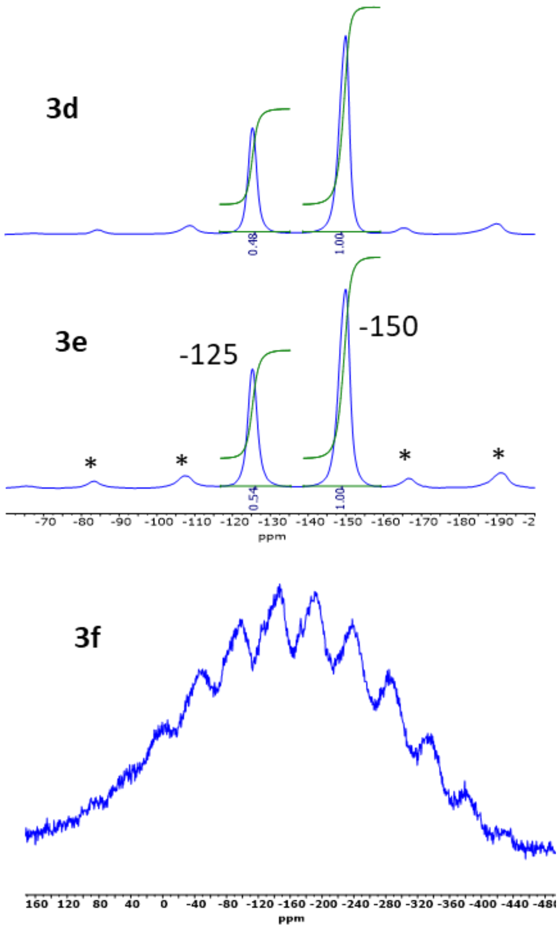

Figure S3: ^{29}Si MAS and CPMAS ssNMR spectrum for **1a-1d**

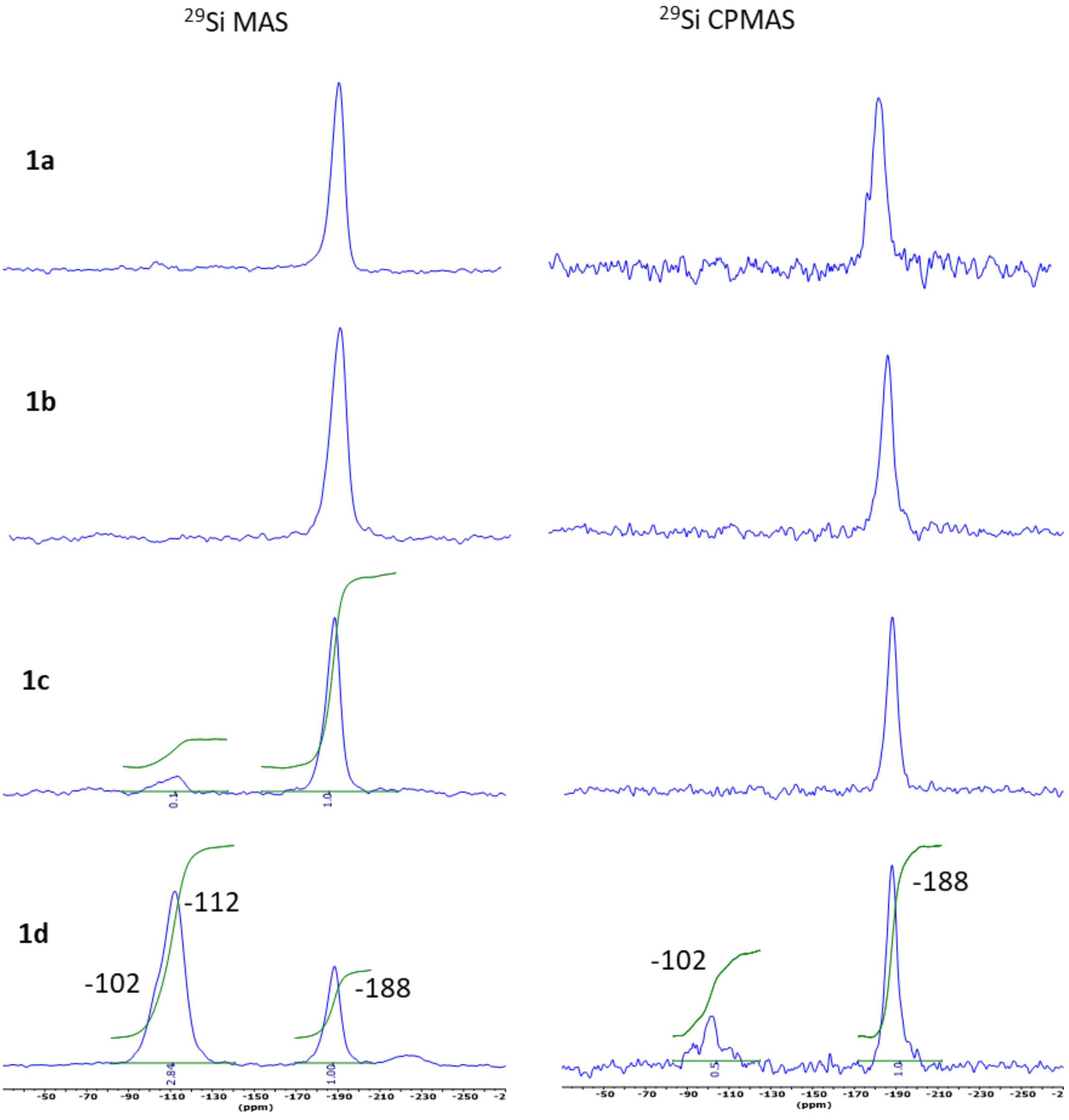

Figure S4: ^{29}Si MAS and CPMAS ssNMR spectrum for **1e-1g**

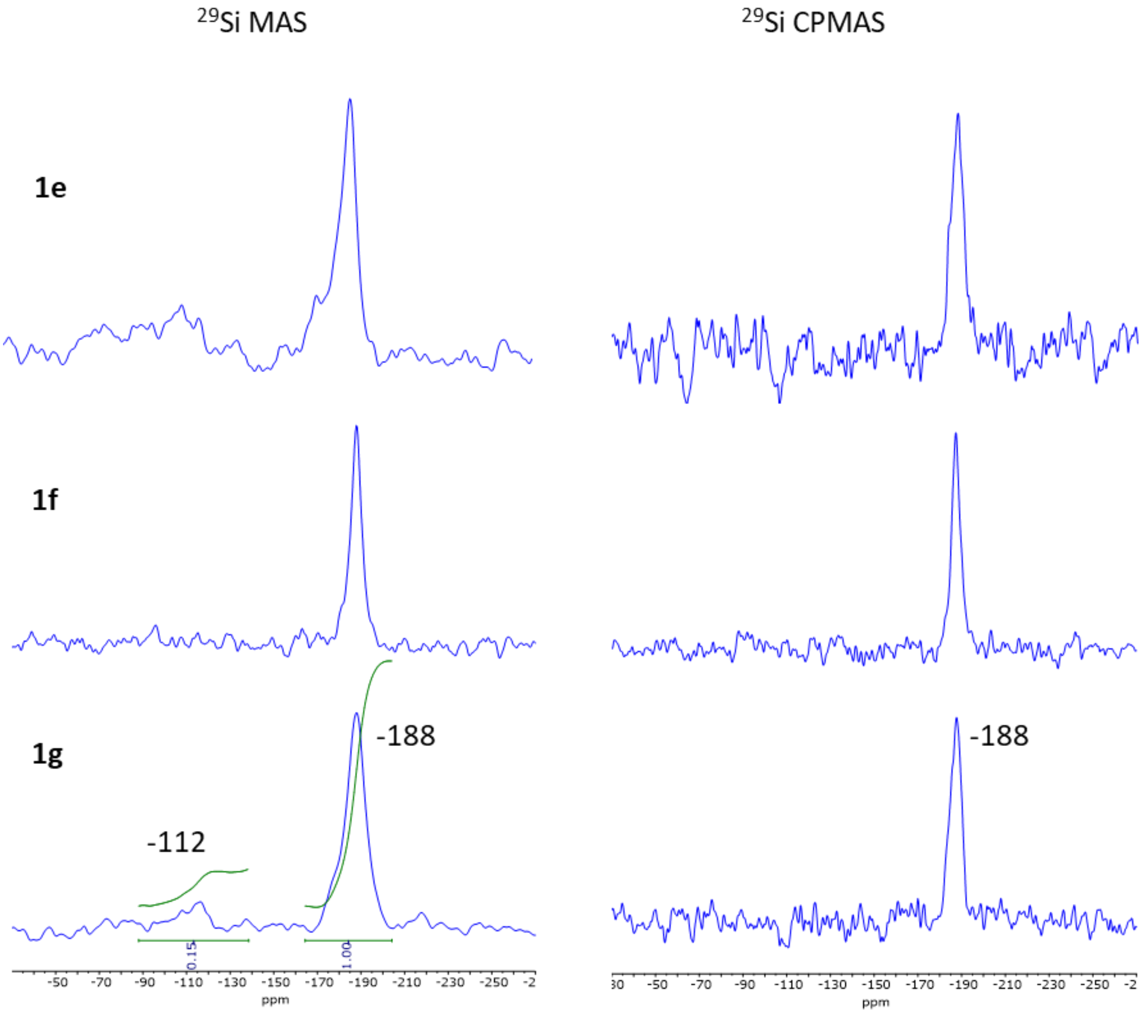

Figure S5: Thermal variation of the optical reflectivity (desolvated sample) for **1e-1g** (effect of the reaction time)

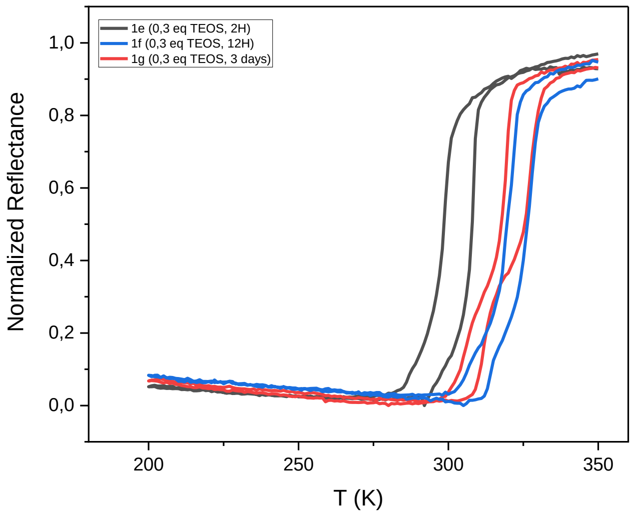

Figure S6: Thermal variation of the optical reflectivity upon 3 consecutive thermal cycles for **1f** (cycling stability after a first stabilizing cycle)

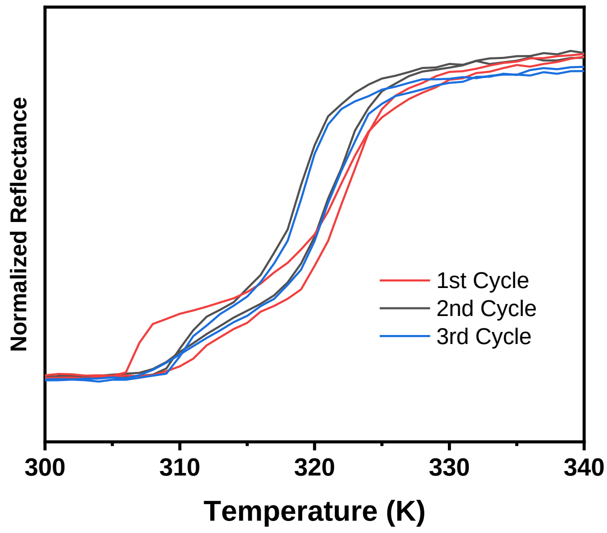

Figure S7: Thermal variation of the powder X-ray diffraction patterns for **3d**

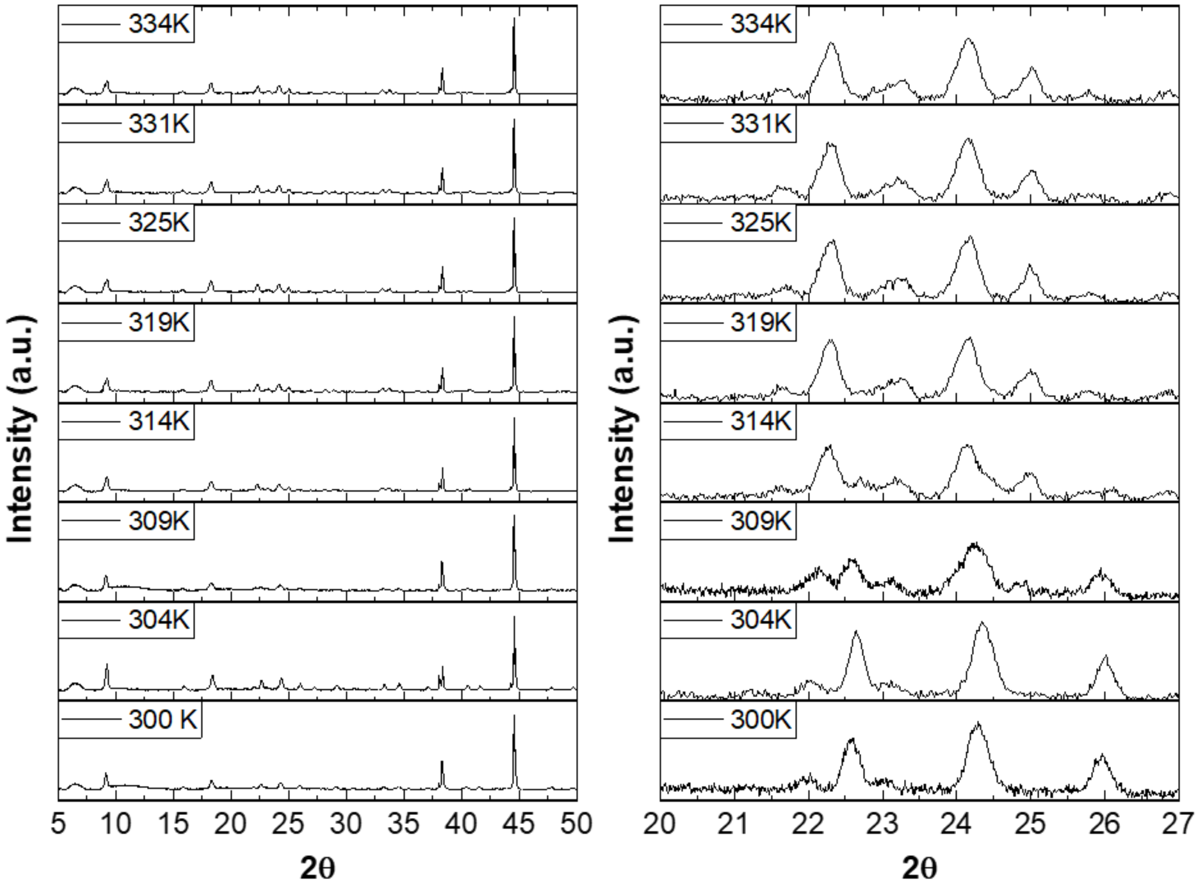

Figure S8: IR spectroscopy and thermal variation of the optical reflectivity for PSM reactions over time

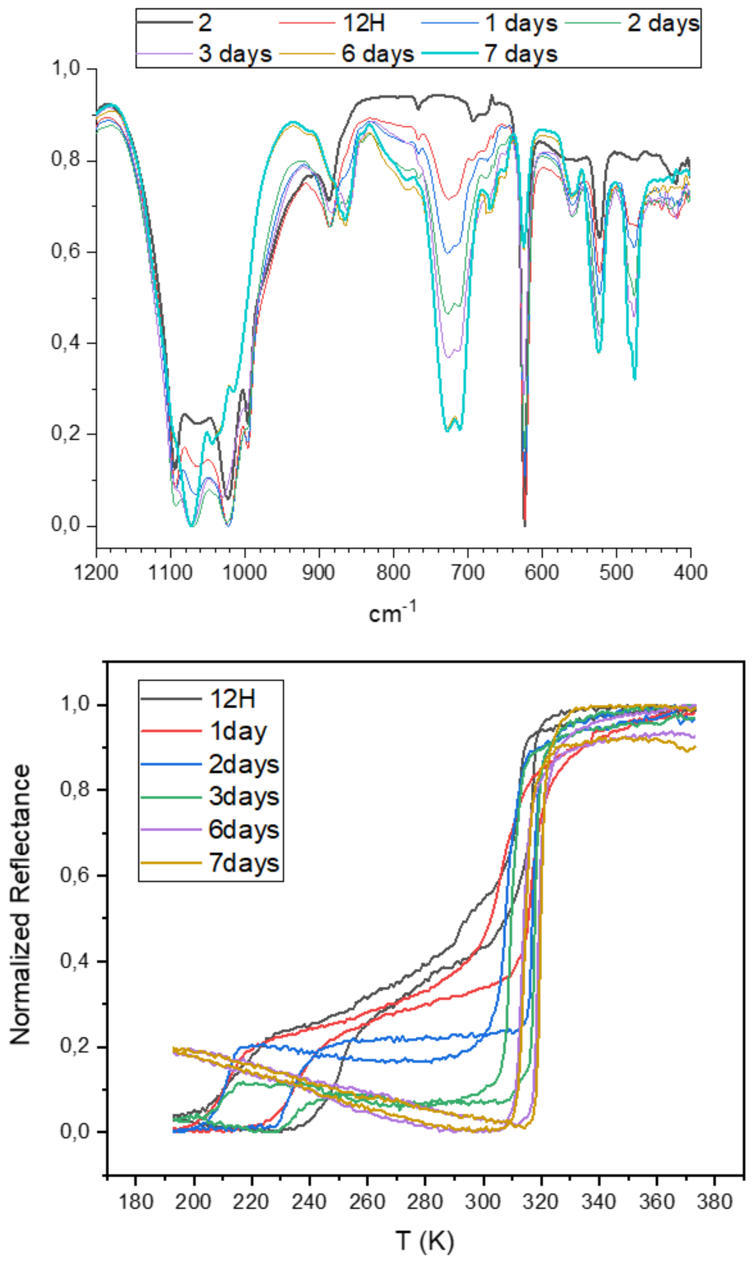

Figure S9: Thermal variation of the optical reflectivity upon 3 consecutive thermal cycles for the sample obtained after 7 days by PSM reaction showing the cycling stability after a first stabilizing cycle

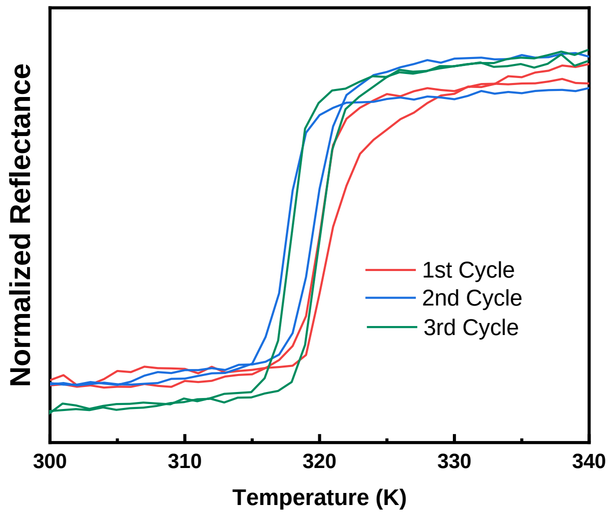

Figure S10: Thermal variation of the optical reflectivity for the desolvated sample obtained after 7 days by PSM reaction just after synthesis and after 1-month storage showing the stability in time

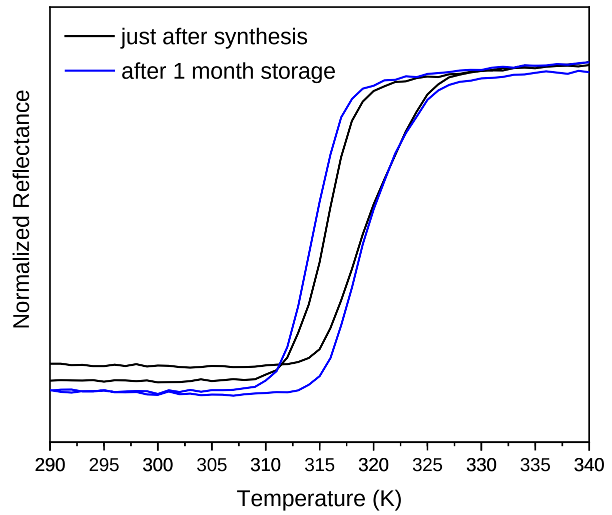

Figure S11: Selection of Transmission Electronic Microscopy images for sample **1a-1g**

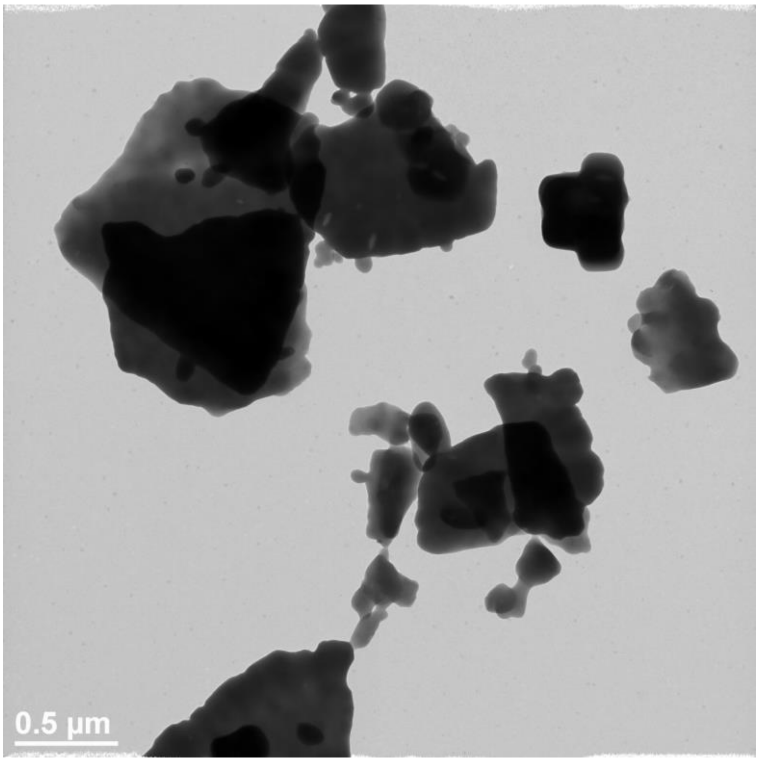

1a (0.125 eq TEOS)

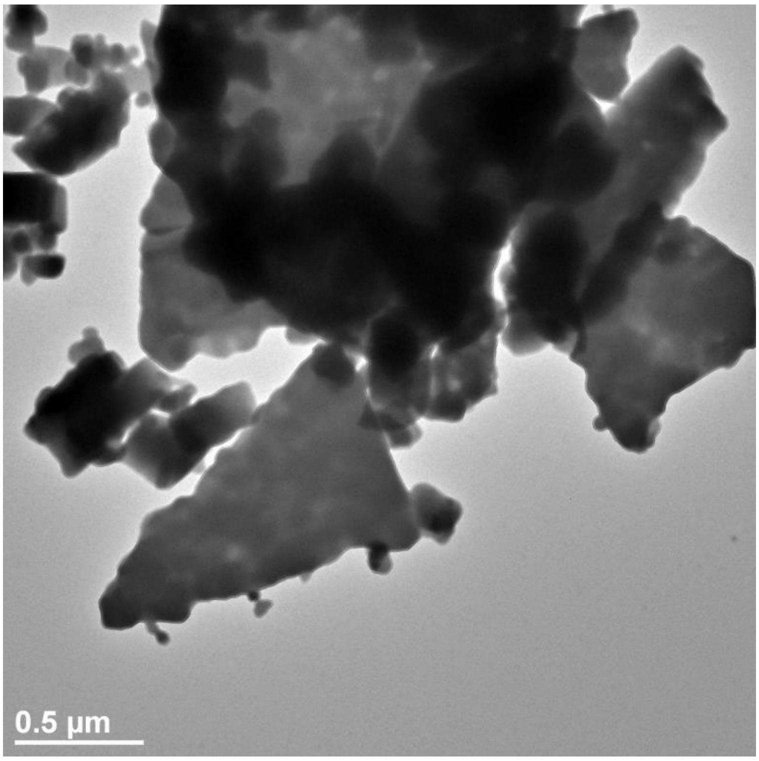

1b (0.25 eq TEOS)

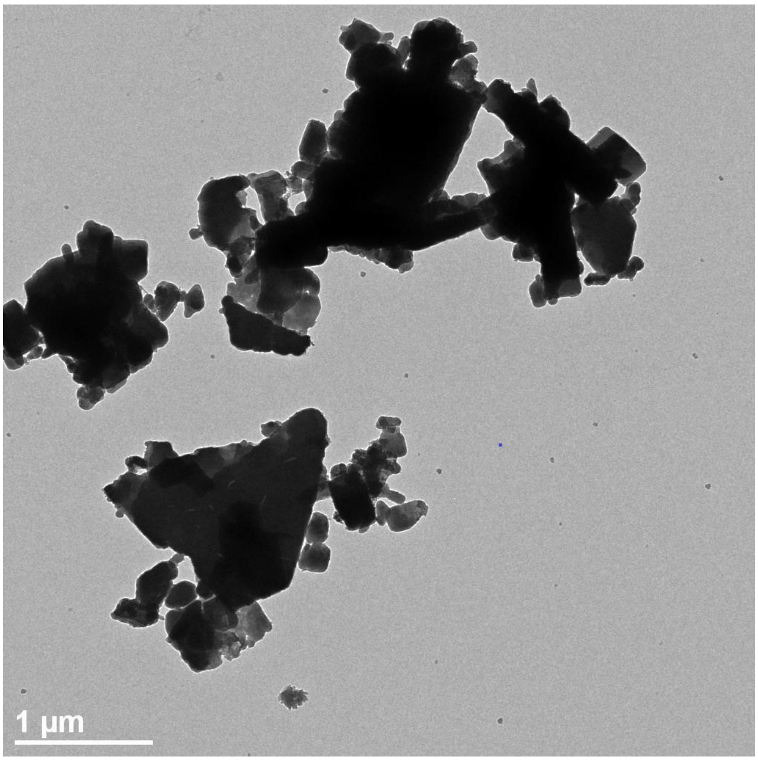

1c (0.5 eq TEOS)

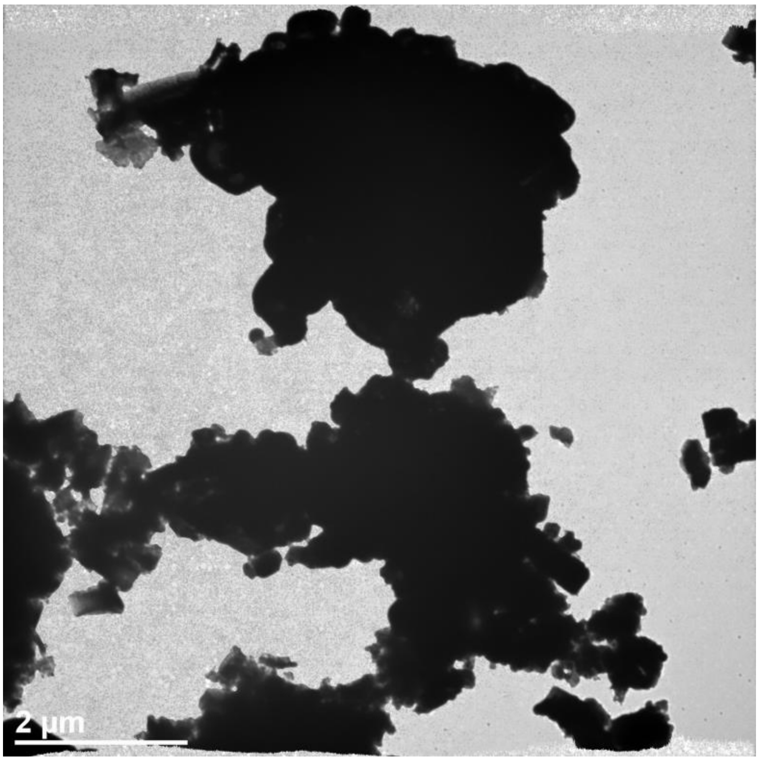

1d (1 eq TEOS)

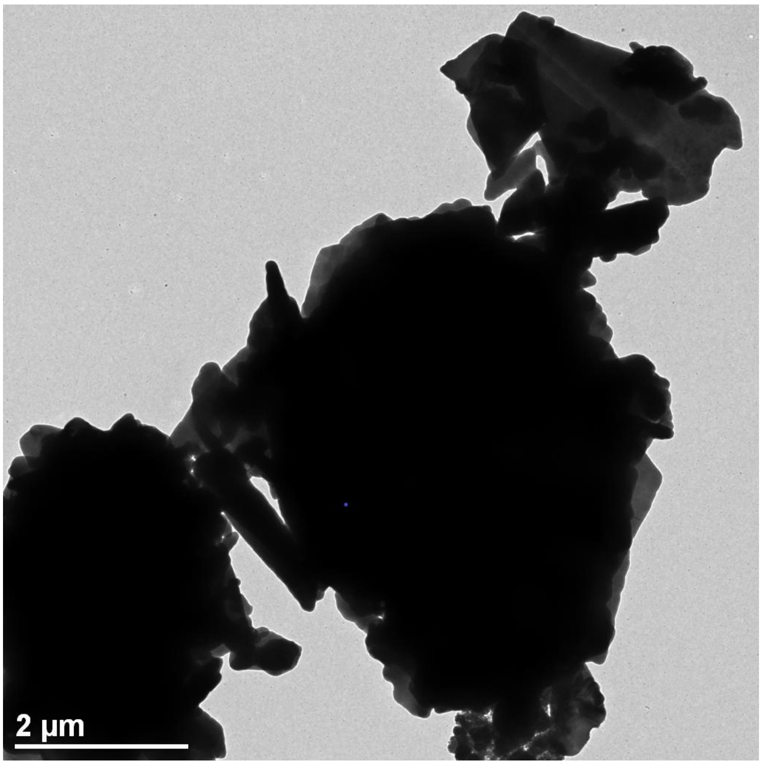

1e (0.3 eq TEOS, 2Hours)

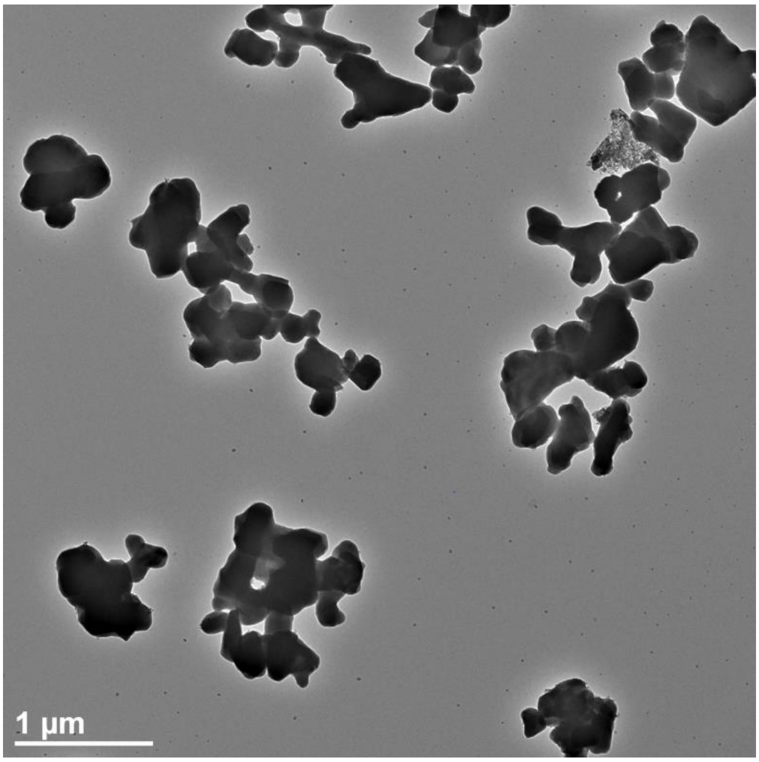

1g (0.3 eq TEOS, 3 Days)

Figure S12: Selection of Transmission Electronic Microscopy images over time for PSM syntheses with 0.3 eq TEOS

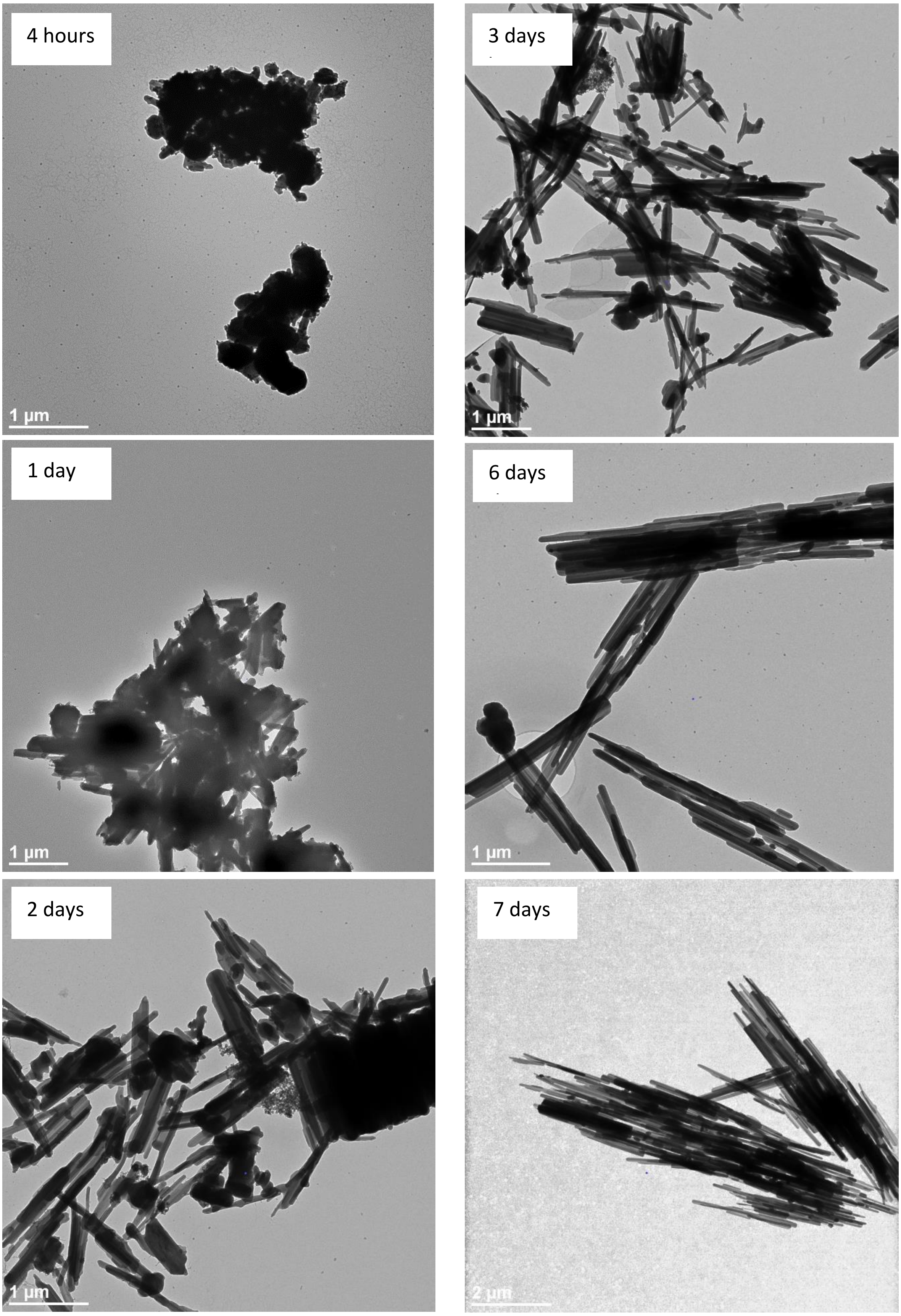

Supplement: DT-053-D4DT00267A-s001 [file DT-053-D4DT00267A-s001.pdf]
